# Evolution of the human tongue and emergence of speech biomechanics

**DOI:** 10.3389/fpsyg.2023.1150778

**Published:** 2023-05-31

**Authors:** Axel G. Ekström, Jens Edlund

**Affiliations:** Division of Speech, Music and Hearing, KTH Royal Institute of Technology, Stockholm, Sweden

**Keywords:** evolution of speech, speech articulation, human evolution, speech production, primatology, articulatory phonetics, coarticulation, speech motor control

## Abstract

The tongue is one of the organs most central to human speech. Here, the evolution and species-unique properties of the human tongue is traced, via reference to the apparent articulatory behavior of extant non-human great apes, and fossil findings from early hominids – from a point of view of articulatory phonetics, the science of human speech production. Increased lingual flexibility provided the possibility of mapping of articulatory targets, possibly via exaptation of manual-gestural mapping capacities evident in extant great apes. The emergence of the human-specific tongue, its properties, and morphology were crucial to the evolution of human articulate speech.

## 1. Introduction

The shape, proportions, and positioning of the human tongue are crucial components of speech biomechanics, accounting for both articulatory and temporal properties of speech acoustics and production. Here, something of the evolution of the organ and its properties is traced via reference comparative anatomy and speech-like behavior in extant non-human great apes (hereafter great apes), and archeological findings of extinct hominid morphology. A cohesive account of speech evolution must take account of both anatomical and neurological properties. In particular, models should seek to include and consider the evolution and properties of the human tongue.

## 2. Properties and morphology

The mammalian tongue is widely considered a muscular hydrostat (Smith and Kier, 1989; Gilbert et al., 2007; Takemoto, 2008), consisting of muscles with no skeletal support and performing hydraulic movements characterized by the property of being largely incompressible from physiological pressures. Functionally, the volume of a muscular hydrostat is constant, and compression in any dimension causes appropriate compensatory expansion in another. Across species, such properties facilitate mastication and swallowing, but in humans, deformation is a crucial component of speech production mechanics also. For speech evolution, thus, vocal anatomy may provide scholars with clues as to the nature of morphological changes that took place throughout human evolution before speech emerged.

The human tongue possesses four major extrinsic and four intrinsic muscles. The extrinsic muscles (originating outside of the organ itself) are: the genioglossus, responsible for forward and downward movement of the tongue (anterior) and forward movement of the dorsal tongue body extending into the pharynx (posterior); the styloglossi, which retract the tongue; the hyoglossus, which depresses and retracts the tongue; and the palatoglossus, which elevates the posterior position of the tongue; and the four intrinsic muscles (attaching only to other muscles in the tongue body) are the superior longitudinal and inferior longitudinal and transverse and vertical muscles. This gross musculature is largely conserved across primates (Swindler and Wood, 1982; Takemoto, 2008) but the human tongue and face contain a higher proportion of slow-twitch myosin fibers, compared to other primates (Sanders et al., 2013; Burrows et al., 2014).

Compared to other mammals, in (adult) humans, the larynx – and therefore also the tongue root, as the larynx is suspended from the basihyoid bone – is permanently retracted downward into the throat (Negus, 1949; Lieberman, 1984, 2012; de Boer and Fitch, 2010; Lieberman et al., 2001). In comparison – as was noted by both Negus (1949) and Crelin (1987) – the tongues of nonhuman mammals, such as sheep, dogs, cats, macaques, spider monkeys, chimpanzees (as well as human infants, who achieve the adult configuration in childhood), are located entirely within the oral cavity. Studies of dissected specimens have since been complemented with studies of live vocalizing animals (Fitch 1997, 2000a; Fitch and Reby, 2001; Weissengruber et al., 2002) illustrating that nonhuman animals typically do not employ their tongues in vocalization. Further, while larynx lowering is found in other species (Fitch and Reby, 2001), these reflect wholly separate adaptations from that of humans. For example, the lowering of the larynx in the Red Deer studied by Fitch and Reby does not – and cannot – markedly change the corresponding phonetic range of the animal.

This is so because, in the words of Lieberman (2012, p. 612): “the larynx transiently descends [in deer] by increasing the distance between the hyoid bone and larynx. This maneuver does not change the shape of the SVT—its cross-sectional area function as a function of distance.” The tongue remains firmly anchored in the mouth of the animal. In comparison, the descended position of the human larynx is part of a suite of extensive anatomical changes in evolution, involving the tongue’s reshaping and partial descent into the pharynx, expansion of the pharyngeal cavity, and restructuring of the cranium (Negus, 1949; Bosma, 1975; Laitman et al., 1978; Laitman and Heimbuch, 1982; Lieberman and McCarthy, 1999). Thus, while Fitch (2000b) claims that a lowered larynx evolved to shift down resonance frequencies and provide impressions of greater size, this claim as applied to humans is based on a false equivalency. The claim is likely true of various nonhuman animals, including Red Deer (Fitch and Reby, 2001); however, the same mechanism does not explain larynx lowering in human vocal tracts. The two are functionally nonequivalent.

**Figure 1:**
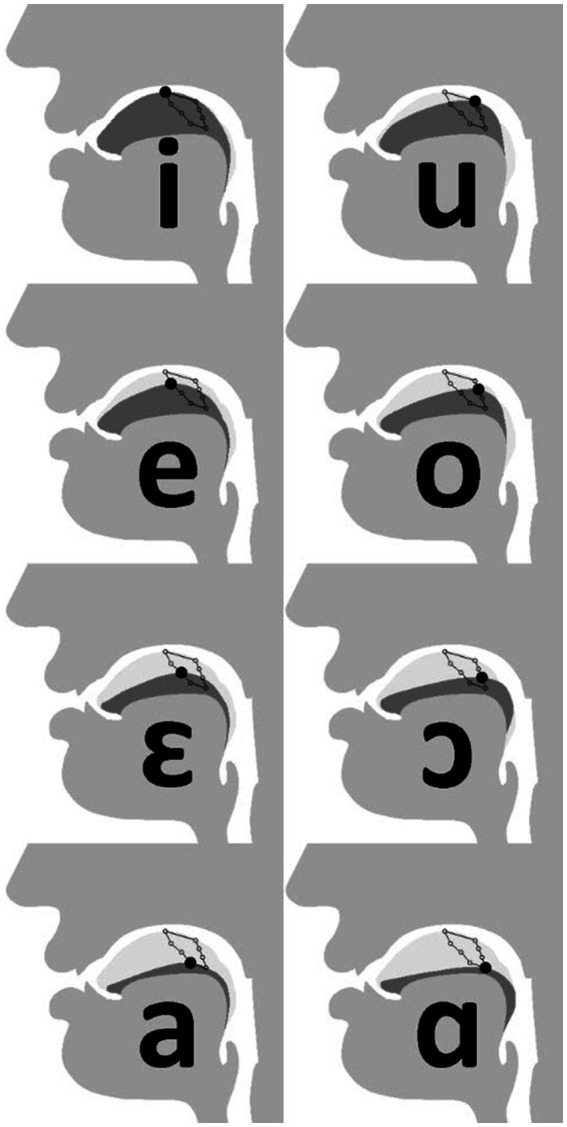
Tongue position for cardinal vowels. [i] (top-left), [u] (top-right) and [a] (bottom-left) are quantal vowels, produced in comparatively stable articulatory space (Lieberman, 1984, 2012; Stevens, 1989). Image adapted from Ishwar (2020). CC BY 3.0.

Through its reconfiguration, the human supralaryngeal vocal tract acquires a roughly 1:1 relationship between horizontal and vertical sections. The human tongue has been rounded, compared to that of the “flatter” tongues of nonhuman mammals (Negus, 1949; Crelin, 1987; Takemoto, 2008; Iwasaki et al., 2019). The resulting flexibility of tongue motion makes possible the production of *quantal* vowels including [i] and [u] (the vowels in “see” and “boot,” respectively), and velar plosives [k] and [g] (the first consonants in “cup” and “good,” respectively) (Lieberman et al., 1969, 1992; Stevens, 1989; Carre et al., 1995; de Boer and Fitch, 2010; Lieberman, 2012). In comparison, ascribing hydrostatic properties to the chimpanzee tongue indicates that its freedom of motion is primarily in protrusion and retrusion, as opposed to deformation dorsally inside the oral cavity (required for a variety of speech sounds) (Takemoto, 2008). Crucially, anterior degrees of freedom are necessary for achieving the full extent of human articulatory space (see, e.g., Engwall, 2003). For example, both [i] and [u] are high vowels, articulated with the tongue tip or body arched toward the palate, respectively (Figure 1). Thus, it is the relative position and shape the tongue, rather than position of the larynx *per se*, which are central for speech (Lieberman, 1984, 2012; Carre et al., 1995; de Boer and Fitch, 2010). No nonhuman mammal have ever been shown to attain the same configuration necessary for the extremities of human speech (Lieberman, 1984, 2012; de Boer and Fitch, 2010; Fitch et al., 2016; Ekström, 2023a).

## 3. Evolutionary history

### 3.1. The primate tongue is deemphasized for food intake

While estimates differ, the lineage leading to modern humans is assumed to have diverged from that of *Pan* around ~7 Mya (White et al., 2009). The phylogenetically more distant Strepsirrhini (e.g., lemurs) possess lingual features markedly different from those of extant haplorrhines (Old World monkeys and apes, and New World monkeys) (Machida et al., 1967; Iwasaki et al., 2019). Strepsirrhine tongues possess a narrow lingual apex, anterior–posterior elongated outline, and developed sublingual (Fleagle, 2013), giving those species significant degrees of freedom outside the oral cavity for manipulation of food stuff. On the other hand, lingual anatomy of haplorrhines indicates a diminished role of the tongue in food uptake specifically, possibly coinciding with the emergence of opposable thumbs used for active manual manipulation of food (Ankel-Simons, 2007; Fleagle, 2013; Iwasaki et al., 2019).

Concurrently, the evolutionary trend of the hominid head, coinciding in phylogenetic history with a dietary shift from raw to processed and/or cooked foods (Wrangham, 2009) shows the emergence of species that spent less time masticating and gestating its food. Prognathia was reduced (the face pulled back toward the cranium), and dentition and the oral cavity were reduced in size, and the tongue reshaped. The position of the hyoid bone, providing the tongue with an osseous base, also shifted in evolution (Lieberman, 2011). For speech, the same sequence of changes seemingly “freed up” the facial muscles, organs, and larger would-be articulatory complex, scaffolding more extensive motor sequence cycles – i.e., complex syllabic speech (MacNeilage, 1998). The homo lineage represents an extreme of the haplorrhine trend, with manipulation and processing of food via tool use facilitating a near-complete outsourcing of food intake processes to the hands (Osvath and Gärdenfors, 2005; Wollstonecroft, 2011; Iwasaki et al., 2019).

### 3.2. Reconstructing speech capacities of extinct hominids

Seminal work on speech capacities of extinct hominids were performed by Lieberman and Crelin (1971) and Lieberman et al. (1972), who developed reconstructions of Neanderthal (*H. neanderthalensis*) vocal tracts. The authors reconstructed the supralaryngeal vocal tract of the La Chapelle-aus-Saints Neanderthal fossil, and simulated by means of a computer program, all possible vocal tract configurations. They found that the resulting vowel space was (1) greater than that estimated for actual chimpanzee vocalizations (which the authors attributed to the chimpanzee possibly lacking crucial neural mechanisms for fully utilizing the phonetic potential of species’ vocal tracts); and (2) like the vowel space of human infants, it did not include quantal vowels [a], [i], or [u], which require extreme 10:1 midpoint discontinuities in the oral tract (Stevens, 1989; Lieberman, 2012).

Throughout the history of research on evolution of human speech-centric anatomy, no series of efforts are more extensive than those of Crelin (1987, 1989); throughout this undertaking, he became “convinced that our development is a résumé of our evolution” (Crelin, 1989, p. 19). Crelin determined that skulls of both australopithecines and *Homo habilis* were essentially “apelike” (Crelin, 1987). Further, based on comparative analysis including the skull of the Taung child – a juvenile *A. africanus* (Dart, 1925) – Crelin also argued that the vocal anatomical ontogenetic development of the genus was also essentially comparable to that of extant apes (see Nishimura, 2005).

Vocal tracts of *H. erectus* were deemed intermediate in form between the apelike vocal tracts of australopithecines, and that of the “modern” human vocal tracts: “The snout, related to relatively large maxillae, coupled with a relatively short robust mandibular ramus indicates that only a part of the posterior third of the tongue was located low enough in the neck to serve as a short anterior wall to the oropharynx” (Crelin, 1987, p. 158). Finally, following a restoration of the “Steinheim skull” of an adult archaic (likely female) human (*H. heidelbergensis*) estimated to around 250–350 Kya, Crelin noted that the fossil skull base was “identical to that of a present-day *Homo sapiens* skull.” He determined that the archaic human represented by the Steinheim skull would have been capable of the full range of human speech sounds. Crelin’s conclusions, then, was that the full extent of modern human speech capacities likely had not evolved until the emergence of archaic modern humans, but that something of the capacity had evolved with *H. erectus*.

Both the original reconstructions by Lieberman et al. (1972) and later works by Crelin (1987) assumed that flexion of the skull base (cranial-base angle) provided a basis for inferring the likely shape of species’ vocal tracts: “A fossil that had a shallow cranial base similar to that seen in living apes and human newborns presumably had a similar vocal tract, while a fossil having a flexed adult human basicranial angle would have had a human vocal tract” (Lieberman, 2007a, p. 45). Such measurements are problematic, however, as the tongue and larynx continue to descend in humans after the ontogenetic point of stabilization of cranial flexure (Fitch and Giedd, 1999; Lieberman and McCarthy, 1999). It is important to note that this evidence had yet to be presented at the time of either the original reconstructions by Lieberman et al. (1972), or the later efforts by Crelin (1987) (see, e.g., Lieberman, 2007a). Nonetheless, it is of note that the central claims made on the basis of those studies – that Neanderthal speech was likely less articulate than that of modern humans, resulting from its not yet having acquired the supralaryngeal airway dimensions that characterize the human condition – are also seemingly supported by other findings, including the observation that fitting a human vocal tract (with a 1:1 relationship between horizontal and vertical sections) to Neanderthal anatomy effectively places the larynx in the chest, a vocal anatomical configuration absent from any existent mammal (see Lieberman, 2007b).

#### 3.2.1. Alternate views

The most widely discussed purported refutation of the findings of Lieberman et al. (1972) is that of Boë et al. (1999), contextually an important work, as it is the only one to couch is suppositions in speech production and acoustics (*cf.* Carlisle and Siegel, 1974; D’Anastasio et al., 2013; Dediu and Levinson, 2013; Frayer, 2017). However, like the earlier reconstructions by Lieberman et al., Boë et al. based their research and argument – that “Neanderthal man was not morphologically handicapped for speech” – on angle of the cranial base (in their work, of a reconstruction of a Neanderthal skull by Heim, 1989). In so doing, however, the authors, while citing the then-recent findings that tongue position and shape could not be inferred from basicranial angle (Fitch and Giedd, 1999; Lieberman and McCarthy, 1999), fail to acknowledge their importance (see Lieberman, 2007a). Boë et al. also fit a human vocal tract to the reconstructed Neanderthal skull. In commenting on this procedure, Lieberman (2007b, p. 552) writes, “The restructuring of the human skull which places the human face in line with the braincase did not take place in Neanderthals, resulting in a long oral cavity. A modern vocal tract placed on a Neanderthal skull would require a tongue displaced down so low into its neck that the creature’s larynx would be in its chest, a configuration absent in any primate species.”

A second source of error in the Boë series of works relates to the “Variable Linear Articulatory Model” (VLAM) procedure, based on an algorithm by Maeda (1990), and consistently employed by Boë et al. throughout their work on the topic. By the logic of this algorithm, research teams led by Boë argued there were no anatomical limitations to Neanderthals’ (Boë et al., 1999, 2002a) or human infants’ (Boë et al., 2002b, 2007) producing the full range of human speech, and that the size of the pharynx was “an irrelevant parameter for speech emergence” (Boë et al., 2002b). Crucially, however, the Maeda algorithm – based on adult human French speakers – maintains the basic shape of the human supralaryngeal vocal tract, even if it results in anatomically impossible vocal tract configurations – as was indeed the case. The problematic application of the Maeda algorithm was outlined by de Boer and Fitch (2010), see also Lieberman (2007a, 2012). After these refutations, no further work using the VLAM procedure have been produced by teams led by Boë.

The reconstructions by Lieberman and Crelin have been criticized at various times and by various researchers. However, much of the debate have not focused on elements of speech production *per se*, but rather on a deeper anthropological consideration of whether Neanderthal should be considered a separate species from *homo sapiens* (*cf.* a subspecies; *Homo sapiens neanderthalensis*). This debate is largely outside the scope of this text and will not be discussed beyond this point.^1^ However, it is important to note that various authors of critiques of the speech-centric work by Lieberman et al. fail to address basic tenets of the relevant anatomical arguments. For example, Dediu and Levinson (2013), quoting Fitch (2009, p. 133) cite the dynamic lowering of the larynx in nonhuman animals as evidence that the “significance of the descent of the larynx … has been overestimated.” It has already been shown (in section “Properties and morphology”), why this is a non-argument: larynx lowering (permanent or temporary) in nonhuman animals is not functionally equivalent to that found in humans. The two are accomplished disparately, and for different purposes (Fitch and Reby, 2001; Lieberman, 2012), and ontogenetic laryngeal descent in humans is part of a suite of anatomical changes facilitating speech capacities, including a restructuring of the cranium and expansion of the pharyngeal cavity. Dediu and Levinson (2013) also uncritically cite the widely discredited modeling work by Boë et al. (1999) as positive proof against the Lieberman claims (for refutations, see de Boer and Fitch, 2010; Lieberman, 2012).

#### 3.2.2. Speaking hyoids?

The hyoid bone constitutes one of the least represented elements in the fossil record, with the only known findings representing *Australopithecus aferensis* (Alemseged et al., 2006), *H. heidelbergensis* (Martínez et al., 2008), and *H. neanderthalensis* (Neanderthals) (Arensburg et al., 1989). Nevertheless, findings provide useful clues to the evolution of the modern human articulatory apparatus. The human hyoid is bar-shaped and positioned below the tongue, under the inferior margin of the mandibular body, while that of extant great apes is bulla-shaped, and positioned anterior to the tongue root (Falk, 1975; Steele et al., 2013). Comparative studies suggest that the hyoid of *A. afarensis* is bulla-shaped like that of extant great apes (Alemseged et al., 2006) (though in the specimen described, the hyoid was preserved beneath the palate, preventing thorough analysis). Meanwhile, general morphology of the *H. heidelbergensis* hyoid bones found at Sima de los Heuses and described by Martínez et al. (2008) (dated to ~530 Kya), shows a transition away from the bulla-shaped hyoid of extant nonhuman hominids, toward the bar-shaped morphology that characterizes the hyoid of modern humans. The authors suggested that such aspects of modern hyoid bone morphology are a derived feature, inherited from a common ancestor of the Neanderthal and modern humans (Arensburg et al., 1989; Martínez et al., 2008). This determination is consistent with Crelin’s (1987) constructions. Crucially for all such work, however, the shape of the hyoid is maintained in human infants and adults, even as the hyoid and larynx descend in ontogenetic development. Thus, as was argued by Lieberman (1999), the shape of the hyoid *per se* does not, and cannot, inform researchers about the length or shape of the vocal tract of extinct hominids (see also Lieberman et al., 1989), and therefore provides only circumstantial evidence with bearing on actual articulation.^2^

#### 3.2.3. Hypoglossal canals

It has been suggested, based on studies of the hypoglossal canal of the occipital bone (cranial nerve XII), which transmits the nerve supplying all intrinsic and (all but one) extrinsic lingual muscles, that mean areas of the hypoglossal canal of humans is significantly larger than that of other extant hominids (Kay et al., 1998). However, DeGusta et al. (1999) showed that hypoglossal canal size was highly variable in humans, with overlap between modern humans, and both extant nonhuman great apes, and australopithecines. The same conclusions were later enforced by Jungers et al. (2003), see also Lieberman (1999). Thus, the current state of research does not support that the shape of hypoglossal canals provides reliable information about species’ speech capacities.

### 3.3. Summary

To date, it has never been convincingly argued that any other species than *homo sapiens* possessed the full range of modern human speech capacities. While individual elements of anatomy do not in isolation provide researchers with the necessary information for inferring species’ speech capacities, holistic interpretation of those elements – including flexure of the skull base, shape of the hyoid bone, and phylogenetic restructuring of facial morphology – suggest a gradual transition from the apelike vocal anatomy of extinct early human ancestors toward that of modern humans, with *H. erectus* appearing as a likely in-between point. Additionally, no evidence presented on Neanderthal potential speech capacities have convincingly argued that the species’ “vowel space was as large of that of modern humans” (Boë et al., 2002a,b). The current state of research tentatively favors Crelin’s (1989, p. 19) interpretation, that the “evolution of the [human vocal] tract occurred … quite recently in time.”

## 4. The tongue in speech and speechlike behavior

### 4.1. A comparative perspective

Possible tongue involvement in great ape articulation is difficult to study via observation alone (Grawunder et al., 2022; Ekström et al., in press), and any procedure typical of phonetics, such as palatography (measurements of tongue position in speech articulation) is not feasibly applicable to non-human subjects. This is crucial, because the likely limitations imposed on species’ articulatory capacities from tongue morphology strongly suggests the relevant vocal tract area functions are unattainable by those species by the same means as by humans (Takemoto, 2008; Lieberman, 2012). Indeed, available evidence indicates that a human tongue is necessary for achieving the superior lingual curvature via decompression of the tongue body against the hard palate that characterizes various human speech sounds (e.g., Engwall, 2003) (Table 1). The work by Grawunder et al. (2022) also do not provide strong evidence for any involvement of the tongue in vocalizations, aligning with previous work on nonhuman animal vocalizations (Fitch, 1997, 2000a,b; see also Ekström, 2022a). The resulting “vowel-like space” is rather suggestive of the possibility that chimpanzees shift resonance frequencies down (into an /u:/-like dispersion) by using the lips, effectively elongating the vocal tract, and narrowing its lip passage (Fant, 1960). For clues to *in-situ* function, however, we may turn to case studies.

**Table 1:** Portions of the human tongue, and their involvement in a variety of speech gestures.

Sound	Articulation	Phonetic transcription	Example of in-word usage
Close back rounded vowel	Pulmonic airflow through vocal tract, where tongue is positioned close to the hard palate, in the posterior oral cavity (“back”).	[u]	“boot”
Close front unrounded vowel	Pulmonic airflow through vocal tract, where tongue is positioned close to the hard palate, in the anterior oral cavity (“front”).	[i]	“see”
Voiceless dental alveolar plosive	Temporary occlusion of pulmonic airflow via the tongue tip making contact with front teeth, and tongue sides making contact with anterior and lateral alveolar ridge.	[t]	“tip”
Voiceless velar plosive	Temporary occlusion of pulmonic airflow via the tongue body making contact with the soft palate, molars, and gum ridge.	[k]	“can”
Voiceless dental fricative	Tongue tip or blade making contact with upper teeth, constricting airflow and causing turbulence.	[θ]	“the”

All gestures involve active manipulation of the tongue.

In one such study of speechlike utterances by Viki the chimpanzee – raised in a human home and explicitly tutored in speech (Hayes and Hayes, 1951) – one of the authors found that, while Viki had seemingly learned an articulatory gesture roughly corresponding to the lexical form “cup,” this sequence was seemingly (as indicated by comparative analysis with a human speaker) realized as a combination of a voiceless fricative, produced in the dorsal oral cavity, and a voiceless bilabial plosive (Ekström, 2023b). That is, humanlike production of the intended sequence or word was seemingly unavailable. Velar plosive [k] (cup) requires substantial maneuverability of the tongue body amounting to a brief but complete occlusion of pulmonary airflow in the oral cavity. Thus, both anatomical and acoustic-phonetic evidence suggests that the chimpanzee is precluded from the articulatory finesse exhibited by modern humans. The chimpanzee tongue is likely incapable of such extreme compression as to make possible the rapid tongue-body plosive speech gestures involved in, e.g., [k] and the rapid changes involved in everyday speech. Further, because the chimpanzee oral cavity is larger than that of humans (Lieberman, 2011), the distance to would-be articulatory targets (Perkell, 1996) is also greater.

### 4.2. Lingual coarticulation: an emergent property

Speech – temporally and in terms of serial organization – is moves between learned gestures. That is, speech gestures are executed continuously, and in natural speech, articulatory organs do not produce any one gesture in isolation. In the words of Farnetani and Recasens (2010, p. 217) “A fundamental and extraordinary characteristic of spoken language … is that the movements of different articulators for the production of successive phonetic segments overlap in time and interact with one another: as a consequence, the vocal tract configuration at any point in time is influenced by more than one segment.” For non-reduced vowels, maneuvers of the tongue root and tongue body necessary for a shift between one vowel to another are likely capped at ~100 ms in humans (Perkell, 1969) – but a vowel reduced from imposition of coarticulatory constrictions (executable without the prior specification of articulatory target positions) may be actualized at still faster rates.

While a number of studies consider the apparent “syntax” of primate calls (Zuberbühler, 2018), such work rarely studies articulatory gestures involved, but the temporal adjacency or connectedness of calls, call types, or acoustic aspects of calls (but see Fitch, 2000a,b; Grawunder et al., 2022). While a central tenant of human speech, and an obligatory component of speech motor behavior, however, the centrality of coarticulation to speech-centric activity is not readily recognized in the broader literature on language evolution. For example, Fitch (2010), in *The Evolution of Language* devotes less than two pages to the subject, arguing that “Coarticulation seems as likely to be an unfortunate byproduct of producing sounds with a massive tongue as a specifically evolved ‘feature’ of the human vocal tract.” In so writing, Fitch addresses claims by Liberman et al. (1967) who argued that coarticulation evolved to meet the demand of perceptual systems. The argument by Liberman and colleagues verges on teleology (explaining phenomena by their function, rather than their ultimate cause), and Fitch (2010) is correct that coarticulation *per se* need not be considered an evolved feature. However, what Fitch calls “an unfortunate byproduct,” is likely the exact opposite.

Temporal reduction of speech sounds also results in the compact transmission of information. This is of enormous benefit for human systems of auditory perception and short-term memory, which are not capable of storing infinite amounts of incoming information (Miller, 1956; Shiffrin and Nosofsky, 1994; Cowan, 2001). Indeed, this was the line of argument that initially prompted Liberman et al. (1967) to develop their argument and corresponding “motor theory of speech perception”. The supposedly “unfortunate” nature of coarticulation, then, in reality reflects a fortuitous advantage of speech production in general, and of the human tongue, which has evolved the capacity for more articulate speech, compared to that of any other extant animal, in particular. Coarticulation – by selective evolution or by happenstance – allows “chunky” speech perception, the perceptual reduction of acoustic features into consonantal “frames” and vowel “content” (MacNeilage, 1998), effectively concentrating a complex stream of sounds into readily perceivable pseudo units (see also Studdert-Kennedy et al., 1998). Seen from such a perspective, coarticulatory phenomena are suggestive of holistic anatomical-neural coevolution of human speech, rather than a phylogenetic emphasis of one over the other (see Lieberman, 2012, 2017; *cf.* Fitch et al., 2016).

### 4.3. Learned speech as reaching and grabbing

Tongue position in phonemic articulation abides by principles of motor equivalence, such that articulatory goals are achievable via compensatory motions (Gay et al., 1981). That is, just as a reaching action of the hand and arm is continually adjusted in execution to compensate for perturbations, so may lingual “reaching” of the tongue (toward an articulatory target) be executed similarly (Moayedi et al., 2021). The neurological mapping and maintenance of articulatory targets are likely facilitated via a basal ganglion-motor cortical network (Graybiel, 2005; Enard, 2011; Alm, 2021; Ekström, 2022b), where the cerebellum is responsible for continual adjustment of fine-motor behavior (Paulin, 1993), including those involved in speech (Ackermann, 2008; Alm, 2021). In modern humans, there has been significant phylogenetic development of subcortical structures including the cerebellum (Baizer, 2014; Guevara et al., 2021). This is consistent with the emerging picture in neurolinguistics, that a distributed network, rather than any one or few language center(s), is responsible for linguistic abilities (Lieberman, 2000; Murdoch, 2001; Dronkers et al., 2007; Friederici and Gierhan, 2013; Ekström, 2022b).

In comparison, chimpanzees are evidently capable of mapping hand gestures. The success of Washoe the chimpanzee, who is reported to have learned to use hundreds of signs (Gardner et al., 1989; Jensvold and Gardner, 2000), compared to Viki, who was claimed to have spoken *four* words (Hayes and Hayes, 1951; Ekström, 2023b), suggests that one differentiating factor is the relative ease of manual-gestural mapping, as opposed to lingual-gestural mapping, where flexibility is apparently more extensive for the first. While a variety of gestural origins accounts of language evolution have posited an evolutionary trajectory from “hand to mouth” (Corballis, 2003), such claims are not made here. The emergent picture, rather, is suggestive of the capacity for motor mapping of novel gestures or (possibly) gestural sequences being present already in the common ancestor of humans and chimpanzees (and possible more ancient still; Cartmill and Byrne, 2010), though this capacity was largely limited to manual gestures, possibly stemming from the flexible and intentional use of gestural communication observed in wild chimpanzees (Hobaiter and Byrne, 2014), and comparatively inflexible vocal anatomy (Negus, 1949; Lieberman, 1984, 2012; de Boer and Fitch, 2010; but see Lameira, 2017; Ekström, 2023b). Crucial for human speech, however, was an extension of these capacities, to increasingly flexible lingual anatomy. Future research may seek to understand the nature of neurological systems that underlie the acquisition of novel gestures – manual and lingual – in the human and nonhuman primate. Such a theory would help explicate common psychological origins of spoken and signed languages, as contingent on unified neurological frameworks.

## 5. Concluding comments

Available evidence from anthropology and acoustic phonetics suggests (1) gradual evolution of central vocal anatomical features crucial to modern human spoken language, possibly beginning with *H. erectus*, and that (2) until such a time that modern human vocal anatomy was achieved, articulate speech was beyond the articulatory capacities now-extinct ancestral hominids. Further, because tongue movements between articulatory targets are learned, a neural mechanism in humans facilitates the mapping of such targets. Comparative research suggests that similar mechanisms may already have been in place in early chimpanzee-like ancestors – but, given species-typical lingual limitations, may have been largely limited to manual gestures. The evolution of the modern human tongue was an essential element of the evolution of human spoken language.

## Author contributions

AE: conceptualization, writing–initial draft, and writing–editing and review. JE: writing–editing and review. All authors contributed to the article and approved the submitted version.

## Conflict of interest

The authors declare that the research was conducted in the absence of any commercial or financial relationships that could be construed as a potential conflict of interest.

## Publisher’s note

All claims expressed in this article are solely those of the authors and do not necessarily represent those of their affiliated organizations, or those of the publisher, the editors and the reviewers. Any product that may be evaluated in this article, or claim that may be made by its manufacturer, is not guaranteed or endorsed by the publisher.

## Footnotes

^1^For example, the arguments by Carlisle and Siegel (1974) are refuted by Lieberman and Crelin (1974); claims by Dediu and Levinson (2013) are challenged by Berwick et al. (2013).

^2^Further, little work to date has been concerned with the movement afforded by the hyoid explicitly (but see Westbury, 1988; Hiiemae et al., 2002).
